# Anaerobic expression of the *gadE-mdtEF* multidrug efflux operon is primarily regulated by the two-component system ArcBA through antagonizing the H-NS mediated repression

**DOI:** 10.3389/fmicb.2013.00194

**Published:** 2013-07-11

**Authors:** Ziqing Deng, Yue Shan, Qing Pan, Xiang Gao, Aixin Yan

**Affiliations:** School of Biological Sciences, The University of Hong KongHong Kong, China

**Keywords:** multidrug efflux pump, acid resistance, anaerobic adaptation, H-NS, ArcBA two-component system, antagonization

## Abstract

The *gadE-mdtEF* operon encodes a central acid resistance regulator GadE and two multidrug efflux proteins MdtEF. Although transcriptional regulation of *gadE* in the context of acid resistance under the aerobic growth environment of *Escherichia coli* has been extensively studied, regulation of the operon under the physiologically relevant environment of anaerobic growth and its effect on the expression of the multidrug efflux proteins MdtEF in the operon has not been disclosed. Our previous study revealed that anaerobic induction of the operon was dependent on ArcA, the response regulator of the ArcBA two-component system, in the M9 glucose minimal medium. However, the detailed regulatory mechanism remains unknown. In this study, we showed that anaerobic activation of *mdtEF* was driven by the 798 bp unusually long *gadE* promoter. Deletion of *evgA*, *ydeO*, *rpoS*, and *gadX* which has been shown to activate the *gadE* expression during acid stresses under aerobic condition did not have a significant effect on the anaerobic activation of the operon. Rather, anaerobic activation of the operon was largely dependent on the global regulator ArcA and a GTPase MnmE. Under aerobic condition, transcription of *gadE* was repressed by the global DNA silencer H-NS in M9 minimal medium. Interestingly, under anaerobic condition, while Δ*arcA* almost completely abolished transcription of *gadE-mdtEF*, further deletion of *hns* in Δ*arcA* mutant restored the transcription of the full-length P*gadE-lacZ*, and P1- and P3*-lacZ* fusions, suggesting an antagonistic effect of ArcA on the H-NS mediated repression. Taken together, we conclude that the anaerobic activation of the *gadE*-*mdtEF* was primarily mediated by the two-component system ArcBA through antagonizing the H-NS mediated repression.

## Introduction

Drug efflux constitutes an important mechanism in bacterial drug resistance. Efflux activity is mediated by a class of membrane protein transporters called multidrug efflux pumps, which actively extrude a variety of cytotoxic substances including antibiotics out of bacterial cells (Li and Nikaido, 2009; Allen et al., 2010). Based on phylogenetic analysis and functional properties, drug efflux pumps of bacteria are classified into five families: the ATP-binding cassette (ABC), the major facilitator (MFS), the multidrug and toxic compound extrusion (MATE), the small multidrug resistance (SMR), and the resistance nodulation division (RND) family (Piddock, 2006). Among them, the tripartite RND pumps are particularly noteworthy in gram-negative bacteria owing to their capability of exporting a broad range of structurally diverse drugs directly to the outside of bacterial cells (Poole, 2008). The three components of RND efflux pumps include cytoplasmic membrane transporter component of RND family, outer membrane factor (OMF) component, and periplasmic component belonging to the membrane fusion protein (Murakami et al., 2006; Piddock, 2006). The constitutive activity of these efflux systems, such as the AcrAB-TolC pump in *Escherichia coli* and the MexAB-OprM and MexXY-OprM systems in *Pseudomonas aeruginosa*, renders the bacteria a low level, intrinsic resistance to a wide range of toxic substances (Poole, 2005).

Previous genome wide studies revealed that there are 20 efflux systems encoded in the *E. coli* K-12 genome (Nishino and Yamaguchi, 2001). However, except for the housekeeping AcrAB-TolC pump, the expression of other 19 pumps is largely inactive under ordinary laboratory growth condition, i.e., at 37°C in rich medium with aeration (Nishino and Yamaguchi, 2001). Increasing evidence suggests that bacterial stress response, i.e., bacterial growth in the adverse environment of their natural ecological niches and human host, induces the expression of specific efflux pumps (Piddock, 2006; Li and Nikaido, 2009; Poole, 2012a). Indeed, our previous studies revealed that MdtEF (previously known as YhiUV), an RND family pump, is activated during the anaerobic growth of *E. coli*, a signature environment of human gut where the bacterium primarily colonizes (Zhang et al., 2011). The pump was shown to expel the cytotoxic indole nitrosative compounds from *E. coli* cells accumulated during the anaerobic respiration of nitrate and thus protect the bacterium from nitrosative stress under this physiological condition (Zhang et al., 2011). Activation of MdtEF under this condition was shown to be dependent on ArcA, the response regulator of the ArcBA two-component system, which is a global regulatory system dedicated to the anaerobic adaptation of *E. coli* (Green and Paget, 2004), but the detailed regulatory mechanism remains unknown.

MdtEF forms an RND type multidrug efflux system with the common outer membrane channel TolC in which MdtF forms the cytoplasmic membrane transporter and is connected to TolC by the periplasmic protein MdtE (Nishino and Yamaguchi, 2002). Over-expression of the pump from a multicopy plasmid has been shown to confer resistance to a broad range of antimicrobial agents such as some β-lactams (e.g., oxacillin, cloxacillin, and nafcillin), macrolide antibiotic erythromycin, as well as doxorubicin, crystal violet, ethidium bromide, rhodamine 6G, tetraphenylphosphonium bromide (TPP), benzalkonium, SDS, deoxycholate, suggesting that MdtEF is a multidrug resistance determinant (Nishino and Yamaguchi, 2001; Nishino et al., 2003; Lennen et al., 2013). Recently, MdtEF was also found to be involved in the efflux of physiological substance fatty acids (Lennen et al., 2013), and their expression can be induced by various of environmental and physiological signals, such as the entry of the stationary growth phase, N-acetyl-glucosomine, indole, as well as the combined environmental challenges of oxygen limitation and acid stress (Hirakawa et al., 2005, 2006; Hayes et al., 2006; Kobayashi et al., 2006), highlighting the physiological relevance of the MdtEF-TolC efflux pump.

*MdtEF* genes are located in the *gadE-mdtEF* operon where *mdtE* is 339 bp downstream of the *gadE* gene (Keseler et al., 2011). Interestingly, the *gadE* gene in this operon encodes a key regulator of the major acid resistance system in *E. coli* which is composed of the glutamate decarboxylase isoenzymes GadA and GadB, and a dedicated glutamic acid/γ-aminobutyrate (GABA) antiporter GadC (Hommais et al., 2004). During bacterial response to acid stresses, GadA and GadB catalyze the decarboxylation of glutamic acid, yielding GABA, which is subsequently exported by GadC in exchange for another molecule of glutamic acid (Foster, 2004). Since the reaction consumes a proton and releases CO_2_, it effectively limits the intracellular acidification during acid stresses, thus plays an important role in the adaptation and survival of *E. coli* in certain host niches, such as the extremely low pH environment (pH = 2) of gastric acid (Foster, 2004). The expression of this system is primarily subject to the control of the acid resistance regulator GadE in the *gadE-mdtEF* operon (Masuda and Church, 2003).

Owing to its significant roles in acid resistance, transcriptional regulation from the *gadE* promoter has been extensively studied. It was demonstrated that the *gadE* promoter encompasses an unusually large 798 bp intergenic region between the *hdeD* and *gadE* gene (Ma et al., 2004), representing one of the eight similarly large intergenic regions in the entire *E. coli* genome (Tjaden et al., 2002). Transcription from at least four starting sites which are located at -21 (T), -124 (T1), -324/-317 (T2), and -566 (T3) relative to the *gadE* start codon have been identified (Ma et al., 2004; Itou et al., 2009; Sayed and Foster, 2009). More than ten transcription regulators including EvgA, YdeO, GadE, TorR, H-NS, PhoP, RpoS, CRP, MnmE, GadX, and GadW have been found to participate in the regulation of *gadE* during various circumstances of acid stresses through at least five different regulatory circuits (Ma et al., 2004; Sayed and Foster, 2009). Involvement of EvgA, YdeO, H-NS, CRP, GadX, as well as a small RNA DsrA in the regulation of the *mdtEF* gene in the same operon has also been reported (Nishino and Yamaguchi, 2002, 2004; Oshima et al., 2006; Nishino et al., 2008a,b, 2009, 2011). However, little is known about the regulation of the *gadE-mtEF* expression in the anaerobic physiological condition of *E. coli* which is the predominant environmental challenge encountered by the *Entericbacteria* family of bacteria when they are being transmitted from the status of free living to human intestinal tract, except for the involvement of the global transcription regulator ArcA as shown in our previous study (Zhang et al., 2011). Moreover, it remains unclear whether the various of regulatory circuits that activate the expression of *gadE* in the context of acid resistance also lead to the expression of the multidrug efflux genes *mdtEF* present in the same operon.

In the current study we set out to investigate the detailed regulatory circuits governing the anaerobic transcription from the *gadE* promoter in *E. coli* and examine its effect on the expression of the multidrug efflux proteins MdtEF present in the operon. We found that unlike the regulatory patterns occurred during aerobic acid stresses, anaerobic activation of the *gadE-mdtEF* operon is primarily mediated by ArcA through its antagonization of the H-NS mediated repression.

## Materials and methods

### Bacterial strains and plasmids

The bacteria strains and plasmids used in this study are listed in Table 1. Gene deletion mutants were constructed either by P1 phage transduction from the Keio collection (Baba et al., 2006) or using the method described by Datsenko and Wanner (2000). All of the constructed strains were verified by colony PCR and DNA sequencing (BGI, Hong Kong). *E. coli* was cultured in Luria Bertani (LB) broth (USB) or M9 minimum medium supplemented with 0.2% glucose (USB), 0.2% casamino acids (CAA) (USB), 4 μg/ml thiamin (Sigma), 0.1 mM CaCl_2_, 1 mM MgCl_2_, 10 μg/ml ferric ammonium citrate (Sigma), and 0.5 μM (NH_4_)_6_Mo_7_O_24_ (Sigma). Without specific indication, exponential phase cells were harvested when A_600_ of cultures reached around 0.3. Antibiotic concentrations used for bacterial culture or colony screening were 100 μg/ml ampicillin (USB), 20 μg/ml kanamycin (USB), or 25 μg/ml chloramphenicol (USB). All other chemicals without specification were purchased from USB.

**Table 1:** **Bacterial strains and plasmids used in this study**.

**Constructs**	**Genotype**	**Source**
**STRAIN**		
MG1655	Wild type *Escherichia coli*	Mettert and Kiley, 2007
AY0416	MG1655 Δ*lacZ::kan*	Zhang et al., 2011
AY0433	MG1655 Δ*lacZ::kan* Δ*arcA::bla*	Zhang et al., 2011
AY0451	MG1655 Δ*arcA::cat mdtE-FLAG::kan*	Zhang et al., 2011
AY0452	MG1655 *mdtE-FLAG::kan*	Zhang et al., 2011
AY1543	MG1655 Δ*gadE ΔlacZ::Km*	This study
AY1603	MG1655 Δ*gadX::cat mdtE-FLAG::kan*	This study
AY1611	MG1655 Δ*rpoS mdtE-FLAG::kan*	This study
AY1612	MG1655 Δ*evgA mdtE-FLAG::kan*	This study
AY1613	MG1655 Δ*crp::cat, mdtE-FLAG::kan*	This study
AY2304	MG1655 Δ*mnmE mdtE-FLAG::kan*	This study
AY2305	MG1655 Δ*ydeO mdtE-FLAG::kan*	This study
AY2306	MG1655 Δ*ydeO* Δ*lacZ::kan*	This study
AY2311	MG1655 Δ*mnmE* Δ*lacZ::kan*	This study
AY2312	MG1655 Δ*evgA* Δ*lacZ::kan*	This study
AY2313	MG1655 Δ*rpoS* Δ*lacZ::kan*	This study
AY2400	MG1655 Δ*hns* Δ*lacZ::kan*	This study
AY2401	MG1655 Δ*crp* Δ*lacZ::kan*	This study
AY2420	MG1655 Δ*hns mdtE-FLAG::kan*	This study
AY2421	MG1655 P*icdA-lacZ* Δ*mnmE*	This study
AY2422	MG1655 P*lpdA-lacZ* Δ*mnmE*	This study
AY2426	MG1655 Δ*hns* Δ*arcA::bla* Δ*lacZ::kan*	This study
AY2442	MG1655 Δ*hns* Δ*arcA::cat* mdtE-FLAG::kan	This study
PK9460	MG1655 P*lpdA-lacZ*	Gift from Prof. Patricia J. Kiley (University of Wisconsin-Madison)
PK9463	MG1655 P*lpdA-lacZ* Δ*arcA*	Gift from Prof. Patricia J. Kiley
PK9483	MG1655 P*icdA-lacZ*	Gift from Prof. Patricia J. Kiley
PK9484	MG1655 P*icdA-lacZ* Δ*arcA*	Gift from Prof. Patricia J. Kiley
**PLASMID**		
pNN387	Single-copy vector, Cm^r^, a NotI-HindIII cloning site upstream of promoterless *lacZ*	Kobayashi et al., 2006
pNN387mdtE	pNN387 (P*gadE-lacZ*)	Nishino and Yamaguchi, 2001
pAY1633	pNN387 (P*mdtE*-*lacZ*)	This study
pAY1655	pNN387 (P2/P4+P1+P*-lacZ*)	This study
pAY1656	pNN387 (P4+P1+P*-lacZ*)	This study
pAY1657	pNN387 (P1+P*-lacZ*)	This study
pAY1658	pNN387 (P*-lacZ*)	This study
pAY2401	pNN387 (P3*-lacZ*)	This study
pAY2402	pNN387 (P3+P2/P4*-lacZ*)	This study
pAY2403	pNN387 (P3+P2/P4+P1*-lacZ*)	This study
pAY2404	pNN387 (P2/P4*-lacZ*)	This study
pAY2405	pNN387 (P2/P4+P1*-lacZ*)	This study
pAY2406	pNN387 (P1*-lacZ*)	This study
pAY2409	pNN387 (P*mnmE-lacZ*)	This study

### Construction of promoter-*lacZ* transcription reporter in pNN387

Promoter*-lacZ* fusions were constructed based on the methods described previously (Zhang et al., 2011). Firstly, DNA fragments corresponding to the desired promoter regions were amplified using iProof High Fidelity DNA polymerase (Bio-Rad). The PCR condition and program is as follows: initial denaturation at 98°C for 30 s followed by 35 cycles of denaturation, annealing, and extension which are achieved by the program of: 98°C for 10 s, 60°C for 30 s, 72°C for 45 s, and the final extension at 72°C for 5 min. The pair of primers used in PCR includes protective bases (lowercase), restriction enzyme site (NotI or HidIII), followed by ~20 bp homologous to the 5′- and 3′-end of the desirable promoter regions respectively (e.g., PgadE-F: 5′-aaggaaaaaaGCGGCCGCTTACCCCGGTTGTCACCCGGAT-3′; PgadE-R: 5′-cccAAGCTTAACTTGCTCCTTAGCCGTTATC-3′). Primers for construction of other *promoter-lacZ* fusions were designed similarly and the detailed sequences are available upon request. The PCR fragments were purified using illustra GFX PCR DNA and Gel Band Purification Kit (GE) followed by NotI/HindIII (NEB) digestion, and then were ligated into NotI/HindIII (NEB) digested single copy plasmid pNN387 using Quick Ligase (NEB). Colonies were screened by chloramphenicol resistance and then verified by colony PCR and DNA sequencing (BGI, Hong Kong).

### Construction of chromosomal *mdtE-flag* by P1 vir transduction

Construction of *chromosomal mdtE-FLAG* in various of deletion mutants was achieved by P1 phage transduction using the lysate from the strain AY0451 (Zhang et al., 2011). To prepare the lysate of the donor strain, AY0451 was grown in 2 ml LB medium till A_600_ achieved 0.1–0.2, and then 40 μl P1vir and 10 mM CaCl_2_ was added to the culture. The mixture was shaken at 37°C for ~3 h or till cells lysed. 2 drops of chloroform per ml of culture was then added to the lysate to diminish any chance of cell viability. After a vortex and centrifugation at 16,000 g for 2 min, the supernatant was transferred to a fresh tube and stored at 4°C in the presence of couple of drops of chloroform. Culture of the recipient strain was prepared as follows: the recipient strain was grown in LB medium till A_600_ exceeded 0.7. 10 mM CaCl_2_ was then added followed by continuing growth for 15 min. 200 μl of the culture was then mixed with 100 μl lysate of the donor strain and incubated at 37°C for 20 min. Following the incubation, 100 μl of 1 M citrate was added to terminate the infection. Following the addition of 600 μl LB medium, the transductant was incubated at 37°C for 1 h before being spread onto LB agar plates containing kanamycin and 4 mM citrate. Desirable colonies were screened by colony PCR and were verified by DNA sequencing (BGI, Hong Kong).

### β-Galactosidase activity assay

β-galactosidase activity assay was performed based on the method by Miller (1972) and detailed experimental procedure has been described previously (Zhang et al., 2011). Briefly, cells were grown anaerobically by inoculating a small number of cell cultures (initial cell density of 10^3^ cells/ml) into screw-capped culture tubes filled with M9 medium, and subsequently cultured in 37°C water bath without aeration. When A_600_ of the cultures reached ~0.3, tetracycline (10 μg/ml) was added to terminate protein synthesis and cell growth, and the cultures were placed on ice until assayed. The assays were performed in triplicate, and results were presented as the mean in either Miller unit or the percentages relative to the activity of full length promoter-*lacZ* fusion in WT (wild type) strain. Error bars indicate the standard deviation.

### Total RNA extraction

8 ml anaerobically grown (the same condition as used in β-galactosidase activity assay) culture of *E. coli* MG1655 was mixed with 1.25 ml ice-cold ethnol/phenol stop solution (5% water-saturated phenol pH 4.5 in ethanol) and placed on ice for 10 min before being harvested by centrifugation at 4000 g for 9 min at 4°C. After removing supernatant, the cell pellet was frozen in liquid nitrogen and stored at −80°C to aid lysis. Cells were lysed by resuspending in 800 μl TE buffer (pH 8.0) containing 1.4 μl 36 kU μl^−1^ lysozyme (epicenter), and then placed in 64°C water bath for 2 min. After incubation, 88 μl 3M NaOAc (pH 5.2) was added to adjust the pH and ion strength of the lysate solution. Subsequently, acid-phenol/chloroform extraction followed by ethanol precipitation was performed to obtain the total RNA following the manufacturer's instruction. To remove trace amount of genomic DNA contamination, the extracted RNA was subject to DNase I treatment using the turbo DNA Free Kit (Amibion). Absence of genomic DNA contamination was confirmed by PCR suing the prepared RNA as template. The quantity of RNA was determined using NanoDrop 2000 (Thermo Scientific).

### RNA ligase-mediated rapid amplification of 5′ cDNA ends (5′ RLM-RACE)

1 μg of RNA which was treated with the turbo DNA Free Kit (Amibion) as above and shown as tight, no smearing band on the agrose gel (following the instructions of the FirstChoice RLM-RACE Kit (Ambion)) was treated with the Tobacco Acid Pyrophosphatase (TAP), ligated to the 5′ RACE adapter, and reverse transcripted with reagents supplied in the FirstChoice RLM-RACE Kit (Ambion) following the manufacturer's instruction. The obtained cDNA was subsequently utilized to perform outer (primer: PgadEout: 5′-TCCAGAAATTTAATCGCTTCTTCATC-3′) and inner (primer: PgadEin: 5′-GTACTCGAGGTGATTATCTTTCAACTGCCAAAAGC-3′) PCR using Fastart Taq (Roche) DNA polymerase. The PCR products were gel band purified using PCR DNA and Gel Band Purification Kit (GE) and cloned into BamHI and XhoI sites of pPK7035 (Kang et al., 2005) followed by DNA sequencing. The first nucleotide being sequenced following the 5′ RACE adapter sequence was determined as the transcription start site.

### Sodium dodecyl sulfate-polyacrylamide gel electrophoresis (SDS-PAGE) and western blot

*E. coli* strains containing chromosomal FLAG tagged MdtE were grown anaerobically (the same condition as used in β-galactosidase activity assay) to log phase (A_600_ = 0.3) in M9 minimal medium supplemented with 0.2% glucose, 0.2% CAA, 4 μg/ml thiamin, 0.1 mM CaCl_2_, 1 mM MgCl_2_, 10 μg/ml ferric ammonium citrate, and 0.5 μM (NH_4_)_6_Mo_7_O_24_. Cells were harvested by centrifugation at 3800 g at 4°C for 10 min. Cell pellet was resuspended in lysis mix (BugBuster reagent (Merck) supplemented with 2.5 mg/mL lysozyme and 10 U/mL DNase I (Invitrogen)). Following the cell lysis, a small fraction of the lysis was subject to the *Dc* Protein Assay kit (BioRad) to measure the concentration of total proteins in the lysate. The volumes of the cell lysis loaded onto the SDS-PAGE were then determined based on the concentrations of each of the samples such that each of the lanes contains the same amount of total protein. The lysis was then heated at 55°C for 25 min prior to being subjected to sodium dodecyl sulfate-polyacrylamide gel electrophoresis (SDS-PAGE). Separated proteins were electrically (Wet/Tank Blotting Systems, Bio-Rad; 110V) transferred to a nitrocellulose membrane (Bio-Rad) for 1.5 h. After blocking with 5% non-fat milk in 1% TBST (*T*ris *B*uffered *S*aline with *T*ween 20, 0.1 M Tris, 0.15 M NaCl, 0.1% Tween 20, and pH was adjusted with HCl to 7.6), the membrane was incubated with monoclonal anti-FLAG antibody (Sigma), and then secondary antibody (goat anti-mouse IgG HRP conjuate, Bio-Rad). After treatment of the membrane by the ECL Plus Western Blotting Detection Reagents (GE health), the protein bands were visualized by X-ray film development. Signals corresponding to MdtE-FLAG proteins were quantified using ImageJ (National Institutes of Health) and are presented as percentages relative to the level of MdtE-FLAG in the wild type strain.

### EB stained electrophoresis gel mobility shift assay (EMSA)

Purification and subsequent *in vitro* phosphorylation of His_6_-ArcA protein was following the method described previously (Bekker et al., 2010). Briefly, *E. coli* strain BL21 transformed with pET-His_6_ArcA were grown in LB to A_600_ of 0.4 before adding 1 mM isopropyl-β-D-thiogalactopyranoside (IPTG) to induce the overexpress of His_6_-ArcA. After grown for additional 2.5 h, cells were harvested by centrifugation. Cell pellet was then resuspended in 4 ml of buffer A (0.5 MNaCl, 20 mMTris-HCl, pH 7.9) containing 1.3 mg/ml lysozyme, 30 g/ml DNase and RNase. Cells were then incubated at room temperature for 30 min followed by sonication to lyse the cells. Cell lysate was obtained by centrifugation at 15,000 g for 30 min and then was subjected to a 1.5-ml Ni-nitrilotriacetic acid–agarose column (Qiagen) equilibrated with buffer A. Following washing the column with 10 ml buffer A containing 10 mM imidazole, His_6_-ArcA was eluted with buffer A containing 50 mM imidazole. Purified His_6_-ArcA protein was phosphorylated by incubating the protein in TEGD buffer (50 mM Tris-HCl [pH 7.5], 0.5 mM EDTA, 10% glycerol) supplemented with 5 mM MgCl_2_ and 50 mM (final concentration) carbamoyl phosphate. The mixture was incubated for 90 min at 25°C, and then the phosphorylated ArcA was used immediately for DNA-binding reaction. To initiate the DNA binding reaction, various concentration of DNA probes and phosphorylated proteins were mixed and subsequently incubated at 37°C for 20 min in EMSA binding buffer (pH 7.2, 20 mM Tris, 50 mM NaCl, 10 mM EDTA, 4 mM DTT, 5% glycerol, 0.5 mg/mL BSA). The reaction mixture was then subjected to 6% non-denaturing polyacrylamide gel electrophoresis in 0.5 × TBE buffer. The polyacrylamide gel was visualized under UV light (254–366 nm) following the staining in 0.5 × TBE buffer containing 0.5 μg/ml ethidium bromide (EB) for 10 min.

## Results

### Anaerobic transcription of *mdtEF* is initiated from the *gadE* promoter

*MdtEF* are located downstream of the *gadE* gene, which encodes a central regulator of the glutamic acid dependent acid resistance system in *E. coli* (Nishino and Yamaguchi, 2002). Previous RT-PCR analysis revealed that *mdtEF* were co-transcribed with *gadE*, thus were annotated in the operon of *gadE-mdtEF* (Hirakawa et al., 2006). However, an EMSA study by Nishino et al. showed that EvgA, one of the regulators that can activate the expression of *mdtEF*, could bind to the intergenic region between *gadE* and *mdtEF*, indicating that this region might serve as a potential promoter of *mdtEF* under certain conditions (Nishino and Yamaguchi, 2002). Thus, we first examined whether this intergenic region (namely P*mdtE*) is involved in the transcription of *mdtEF* under anaerobic condition. To address this, we constructed *lacZ* fusion of the intergenic region (denoted as P*mdtE*-*lacZ*) in the single copy plasmid pNN387 and performed β-galactosidase activity assay. It was shown that no transcription from P*mdtE-lacZ* was detected under either aerobic or anaerobic condition, while transcription from P*gadE-lacZ* constructed in the same vector was significantly induced under anaerobic condition (data not shown). These results suggested that anaerobic transcription of *mdtEF* was primarily initiated from the *gadE* promoter. Therefore we focused on the 798 bp P*gadE* region in the following investigation to examine its contribution to the expression of *mdtEF* under anaerobic condition.

### Transcription from the same four starting sites of *gadE* were identified in the anaerobically grown *E. coli*

In previous studies, three transcription starting sites, located at -124 (T1), -324/-317 (T2), and -566 (T3) relative to the *gadE* start codon respectively, have been identified in *E. coli* grown in LB glucose to early stationary phase (A_600_ = 1.75) (Sayed and Foster, 2009) and one transcription starting site located at the -21 position was detected from *E. coli* grown in the EG minimal buffer at pH 5.5 (Ma et al., 2004). To examine whether transcription from all these starting sites, and whether transcription from additional starting site, is active under anaerobic growth condition, we performed 5′ RLM-RACE using RNA isolated from *E. coli* grown anaerobically in M9 glucose medium which has been shown to significantly induce the expression of MdtEF in our previous study (Zhang et al., 2011). 5′-RACE revealed that transcription from the three starting sites identified previously was detected from several clones (data not shown). Transcription starting site of -21 was identified from one clone, but a weak -35 and -10 element was present upstream of this starting site. Two putative new transcription starting sites were also detected, however, since no recognizable -35 and -10 elements were present upstream of the sites, they are probably the digestive products from the established four transcripts. This result suggested that transcription from all four starting sites of *gadE-mdtEF* operon, -21 (T), -124 (T2/T4), -566 (T3), is active under anaerobic conditions. We next investigated the transcription regulatory mechanism of *gadE-mdtEF* from all these sites under anaerobic condition.

### The P3 region is important in the anaerobic activation of the *gadE-mdtEF* operon

**Figure 1:**
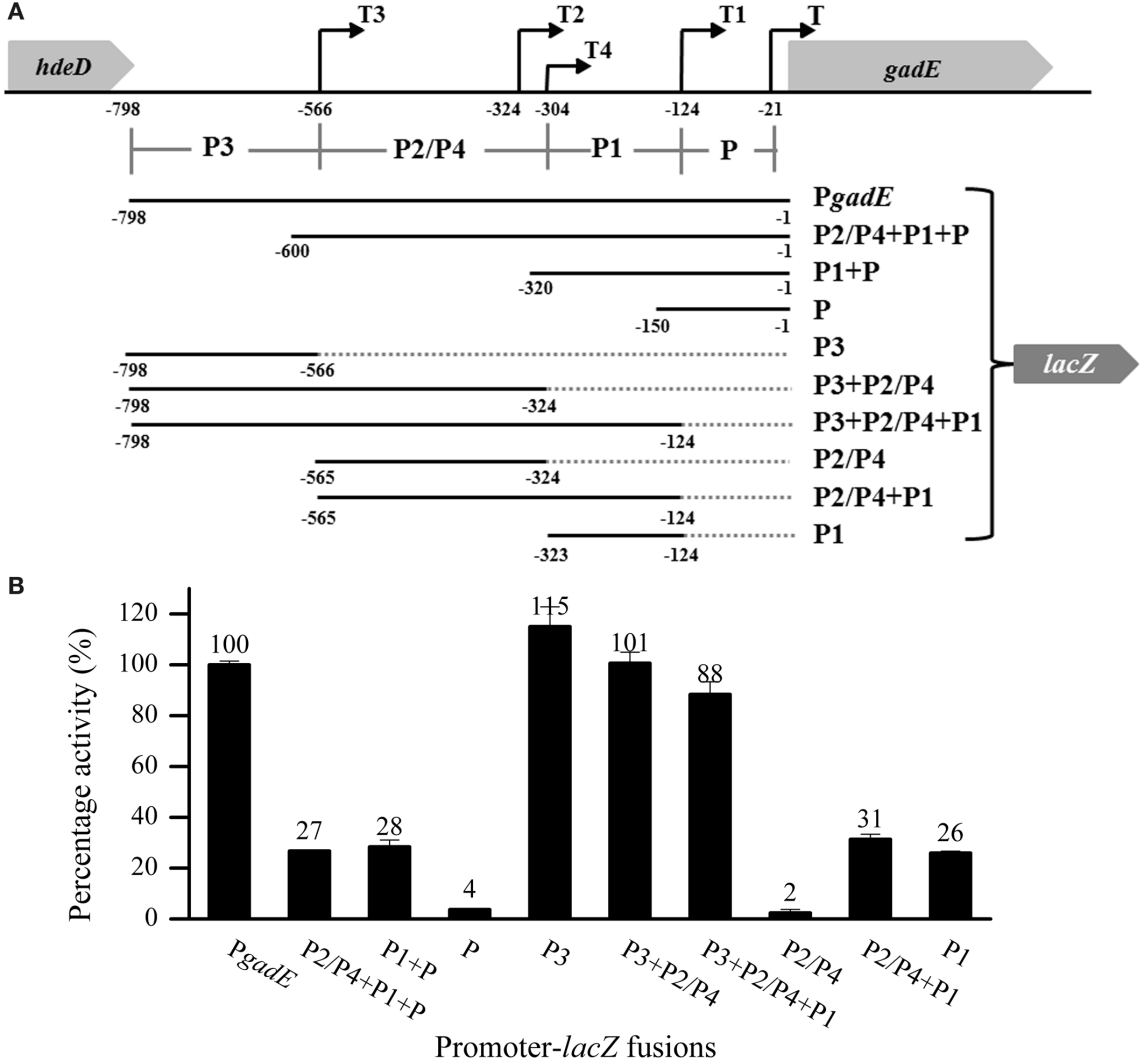
**The P3 region is important in the anaerobic up-regulation of *gadE-mdtEF*. (A)** Schematic diagram of the constructed promoter-*lacZ* fusions. P*gadE* is defined as the 798 bp intergenic region between the *hdeD* and *gadE* genes. Labeled numbers indicate the nucleotides positions relative to the ATG start codon of *gadE*. Transcription starting sites are indicated as black arrows, and the regions upstream of each of the transcription starting sites are designated as its individual promoters, labeled as P3, P2/P4, P1, and P, respectively. **(B)** Promoter activity of all P*gadE* truncations determined by β-galactosidase activity of corresponding *lacZ* fusions. Cells were grown anaerobically to exponential phase (A_600_ = 0.3) in minimal M9 glucose medium. Results are presented as percentages relative to that of full length *gadE* promoter activity (100%), and error bars represent the standard errors of triplicate experiments (*n* = 3).

Following the designation of the P*gadE* region in previous studies (Sayed and Foster, 2009), the four transcription starting sites from 5′- to 3′-end of the 798 bp intergenic region between *hdeD* and *gadE* are designated as T3, T2, T4, T1, and T, respectively (Figure 1A) (Ma et al., 2004; Itou et al., 2009; Sayed and Foster, 2009), and the promoter regions immediately upstream of these transcription start sites are designated as P3, P2, P4, P1, and P accordingly. To examine which promoter regions are important for the anaerobic transcription of the *gadE-mdtEF* operon, we first constructed three P*gadE* truncations which lacked P3, P2/P4, P1 from 5′-end of the region successively, resulting in the promoter-*lacZ* fusion containing the intact P2/P4+P1+P, P1+P, and P regions, respectively (the upper four constructs in Figure 1A). Transcription activity assay showed that promoter-*lacZ* fusions lacking the P3 region, i.e., *lacZ* fusions of P2/P4+P1+P, and P1+P, displayed only ~1/3 activity of that of the full length P*gadE*-*lacZ* fusion (Figure 1B), and that of P region showed almost no activity. These results suggested that the P3 region is important in anaerobic activation of the *gadE-mdtEF* operon and that transcription from the P promoter region is not active under this condition. This result also indicated that the -21 transcription starting site (driven by the P promoter) detected from the RACE analysis might be a digestive product from the transcripts initiated from the other three sites.

To dissect the contribution of each of these promoter regions, we performed another series of truncation on P*gadE*, resulting in *lacZ* fusions containing individual or combined promoters (the lower six constructs in Figure 1A). Transcription activities of these truncations showed that promoter-*lacZ* fusions containing the P3 region, i.e., P3, P3+P2/P4 and P3+P2/P4+P1, displayed similar or even higher activity than that of the full length, whereas promoter-*lacZ* fusions lacking the P3 region, i.e., P2/P4, P2/P4+P1, and P1, displayed very low activities (Figure 1B), confirming the important role of the P3 region in the up-regulation of *gadE-mdtEF* operon under anaerobic condition. This result indicated that a strong activator binding site may exist at the P3 region. Furthermore, transcription of *lacZ* fusion containing the P2/P4 promoter region was almost undetectable, suggesting that similar as the transcription starting site T which is driven by the individual P promoter region, transcription from the individual P2/P4 promoter is also inactive under anaerobic conditions. These results combined explained the observed very low activities of the promoter-*lacZ* fusions of P2/P4+P1, P1, P2/P4+P1+P and P1+P (Figure 1B).

### Involvement of different regulators in the anaerobic expression of *mdtEF* from that of aerobic condition

Previous studies focusing on the expression of *gadE-mdtEF* operon during acid resistance have revealed that several regulators, such as EvgA, YdeO, MnmE, GadE, GadX, CRP, and H-NS, are involved in the regulation of *gadE-mdtEF* operon (Gong et al., 2004; Ma et al., 2004; Hirakawa et al., 2006; Tramonti et al., 2008). However, all those studies were carried out under aerobic condition and primarily focused on the transcription of the *gadE* gene. A previous study from our group focusing on the expression of the multidrug efflux genes *mdtEF* present in the same operon showed that under anaerobic condition the global regulator ArcA activates the expression of *mdtEF*, since Δ*arcA* caused significant decrease of both transcription and protein levels of *mdtE* and *mdtF* (Zhang et al., 2011). However, whether other regulators also participate in the regulation of *mdtEF* under this condition is unknown. Thus, in the present study, we first examined whether previously identified regulators, such as EvgA, YdeO, MnmE, GadE, GadX, and H-NS, also participate in the anaerobic activation of *mdtEF*. Since our previous finding on the anaerobic activation of *mdtEF* occurred in M9 glucose minimal medium in which the effect of CRP on gene expression is minimal, we focused on the effect of other regulators in the current study.

**Figure 2:**
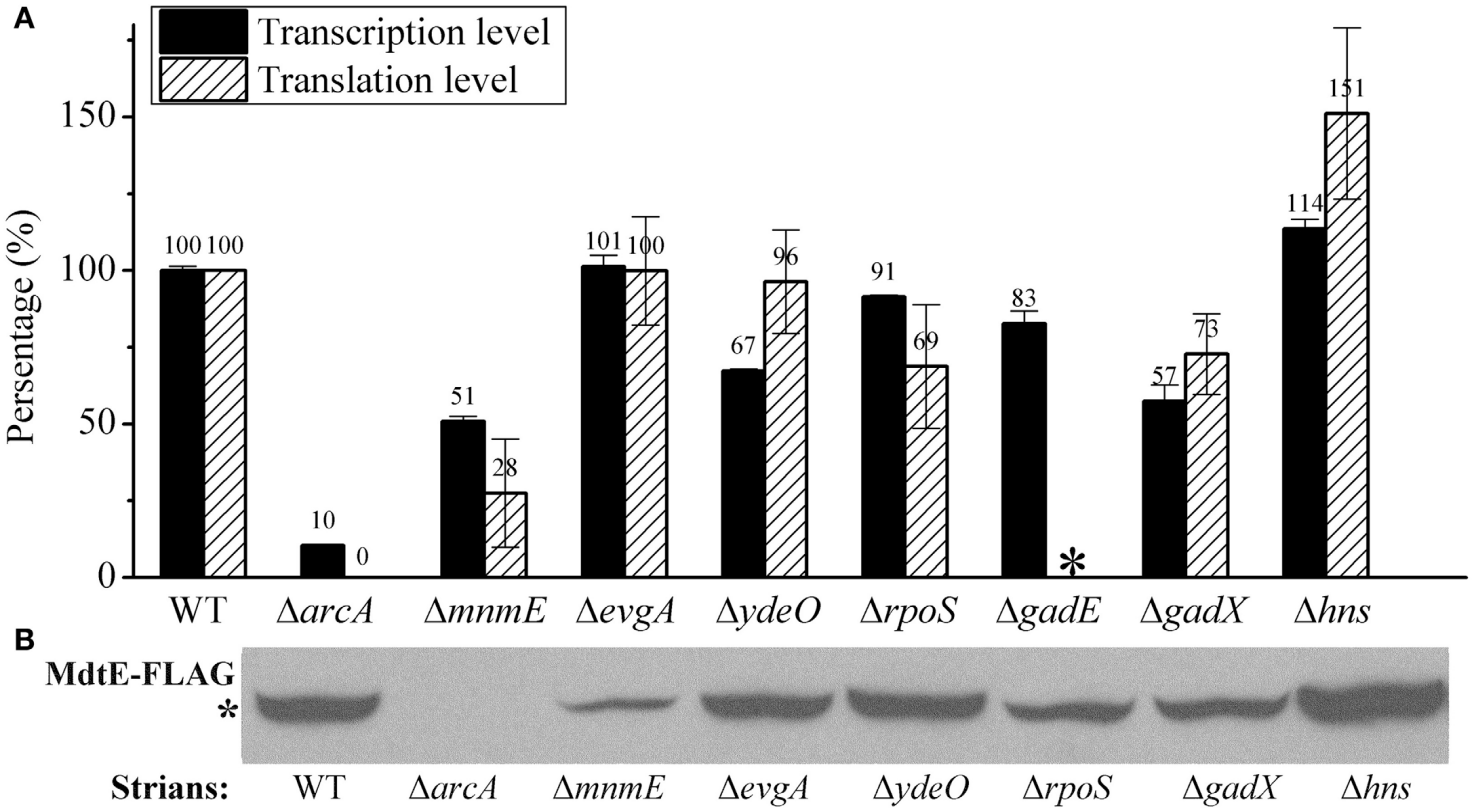
***MdtEF* expression in various mutants at both transcription and translation level under anaerobic condition. (A)** Transcription of *gadE-mdtEF* was determined by β-galactosidase activity of P*gadE-lacZ*, and translation of the gene is determined by Western blot to detect the production of MdtE-FLAG. **(B)** Production of chromosomal MdtE-FLAG in various mutants. Cells were grown anaerobically to exponential phase (A_600_ = 0.3) in minimal M9 glucose medium. All results were presented as percentages relative to the transcription of P*gadE-lacZ* or production of MdtE-FLAG in wild type strain. Error bars represent the standard errors of triplicate experiments (*n* = 3). ^*^mdtE-flag in Δ*gadE* mutant was not constructed because of the close proximity of *gadE* and *mdtEF* which is not applicable by the P1 phage transduction.

We constructed strains containing deletion of each of these genes and measured β-galactosidase activities of P*gadE-lacZ* in these strains. As shown in Figure 2A, deletion of *arcA* caused dramatic decrease of the β-galactosidase activity of P*gadE*-*lacZ*, confirming the role of ArcA in activating the *gadE*-*mdtEF* expression under anaerobic condition. Interestingly, except for *mnmE*, deletion of the regulators previously shown to participate in the regulation of *mdtEF* under aerobic condition, such as EvgA, YdeO, GadE, and GadX, caused no or only a moderate decrease (17–40%) of the transcription of P*gadE*-*lacZ*, and deletion of H-NS caused a moderate increase of *PgadE-lacZ* activity, suggesting that these regulators are not responsible for the activation of *mdtEF* under anaerobic condition. Interestingly, deletion of the GTPase MnmE caused a significant decrease of the transcription of *gadE*-*mdtEF*, suggesting that MnmE may also contribute to the activation of *gadE*-*mdtEF* under this condition. Since the current study focuses on the regulation of the multidrug efflux genes *mdtEF* present in the same operon, we next examined whether deletion of these genes caused alteration of MdtEF protein levels. We constructed chromosomal *mdtE-flag* in these strains (except for Δ*gadE*) and performed Western blot to examine the level of MdtE-FLAG. As shown in Figure 2B, production of MdtE-FLAG in the corresponding deletion mutants showed a similar pattern as that of β-galactosidase activity assay except Δ*mnmE*, which caused a more significant reduction of MdtE expression at translational level. Together these results confirmed that ArcA and MnmE are primarily responsible for the anaerobic activation of *mdtEF*.

**Figure 3:**
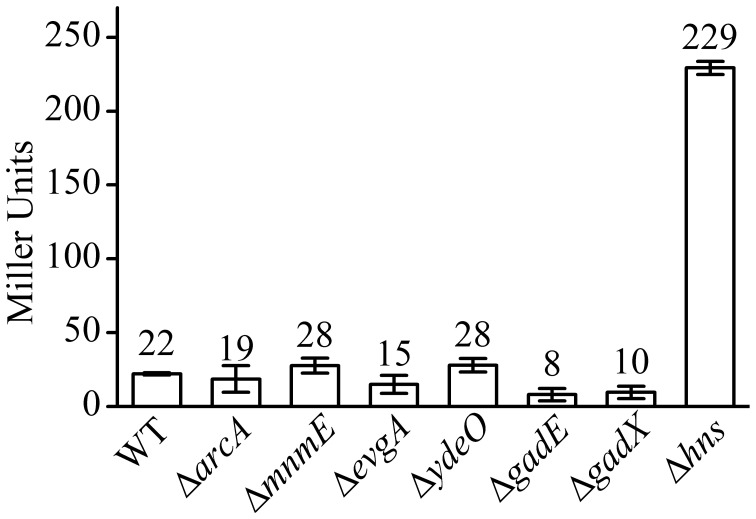
**β-galactosidase activity of P*gadE-lacZ* fusion in WT (MG1655 Δ*lacZ*), Δ*arcA*, Δ*mnmE*, Δ*evgA*, Δ*ydeO*, Δ*gadE*, Δ*gadX*, and Δ*hns* mutant strains under aerobic condition**.

As a control, we also measured the effect of deletion of these regulators on the aerobic expression of *gadE-mdtEF* in *E. coli* grown in M9 glucose medium at log phase. As shown in Figure 3, P*gadE-lacZ* activities in wild type strain, and in Δ*arcA*, Δ*mnmE*, Δ*evgA*, Δ*ydeO*, Δ*gadE*, Δ*gadX* mutants are very low (around 20 Miller units), consistent with the fact that ArcA is not active under this condition. However, in Δ*hns* mutants, the P*gadE-lacZ* activities increased more than 10-fold to about 250 Miller units, suggesting H-NS strongly represses the expression of *gadE-mdtEF* under aerobic condition in the M9 glucose minimal medium. This result is consistent with previous findings that H-NS represses the transcription from P*gadE* through its interaction with various sites in the *gadE* promoter region (Sayed and Foster, 2009). It is noted that deletion of *evgA*, *ydeO*, *gadE*, and *gadX* did not show obvious effect on the transcription of P*gadE-lacZ* in this assay. This is not unexpected as previous studies on the regulation of *gadE-mdtEF* by these regulators was found to be effective in the acidified minimal medium (pH 5.5) containing glucose or during stationary phase in rich medium (Ma et al., 2004), whereas the current study is carried out in the neutral M9 minimal medium with glucose, the identical culture medium as in our previous assay which showed increased expression of MdtEF under anaerobic condition. These results together suggested that the regulatory network of *mdtEF* expression under anaerobic condition is different from that under aerobic condition, and anaerobic activation of *mdtEF* is largely dependent on ArcA and MnmE.

### Deletion of EvgA, YdeO, H-NS does not have obvious effect on the transcription from individual promoters under anaerobic condition

Our investigation above showed that ArcA and MnmE are the major activators responsible for the expression of *gadE-mdtEF* under anaerobic condition, whereas other regulators tested did not show obvious regulatory effects. However, since four promoter regions (P, P1, P2/P4, and P3) are involved in the transcription initiation of *gadE-mdtEF*, we cannot rule out the possibility that they might participate in the regulation of individual promoters contained in the complete 798 bp promoter of *gadE*. For example, it is possible that a certain regulator activates the transcription from one promoter region while represses transcription from another promoter, consequently cancels its effect on the transcription from the full promoter in our assay. To verify this, we examined the anaerobic transcription activity of those P*gadE* truncations in the deletion mutants constructed in this study except for GadX and GadW since they were indicated to be active in low pH, complex medium or stationary phase (Ma et al., 2003). It was shown that deletion of *evgA*, *ydeO*, and *hns* did not cause significant changes on the transcription activity from each of the individual promoters (data not shown), indicating that these regulators had no effect on the transcript from the individual promoters contained in the 798 bp full promoter of *gadE* either under anaerobic condition.

### GadE autoregulates transcription from P1 region under anaerobic condition

**Figure 4:**
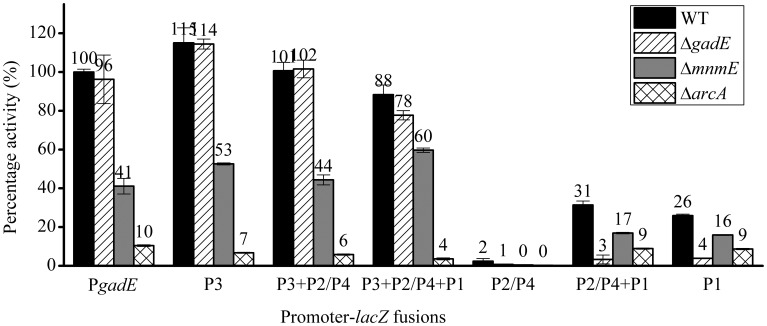
**β-galactosidase activity of P*gadE* truncations in WT (black bars), Δ*gadE* (stripe bars), Δ*mnmE* (gray bars) and Δ*arcA* (grid bars) strains under anaerobic growth condition**.

Interestingly, deletion of *gadE* caused different effects on the transcription from different promoters contained in the full 798 bp P*gadE* region, which is dissimilar from that observed in the case of Δ*evgA*, Δ*ydeO*, and Δ*hns*. As shown in Figure 4 (stripe bars), while *gadE* deletion had no significant effect on the transcription from the *lacZ* fusions of the full length, P3, P3+P2/P4, as well as P3+P2/P4+P1, deletion of *gadE* caused almost complete abolishment of transcription from the P2/P4+P1 and the P1 promoters. This observation combined with the results above which showed that individual P2/P4 was not active under anaerobic conditions suggested that GadE autoactivates the transcription of *gadE-mdtEF* from its P1 promoter. This result is consistent with a previous truncation study performed under aerobic condition, where deletion of *gadE* caused decrease of transcription from P2/P4+P1 and P1 promoters (Sayed and Foster, 2009), and the presence of a conserved GAD box to which GadE binds in the P1 region (Ma et al., 2004). Moreover, the fact that deletion of *acrA* and *mnmE* caused decrease of transcription from P1 at variable degrees whereas deletion of *gadE* almost completely abolished transcription from this promoter suggested that activation of P1 is dependent on the production of GadE which is transcribed from the ArcA and MnmE activated other promoters, such as P3, in the case of the full length, native promoter in the cell.

### ArcA directly binds to multiple sites at the *gadE* promoter to activate the expression of the *gadE*-*mdtEF*

Since ArcA and MnmE were identified as transcription activators of the *gadE-mdtEF* operon, we next asked how these two regulators activate *gadE* expression, i.e., through which promoters these two regulators affect the transcription of the *gadE-mdtEF* operon under anaerobic conditions. To address this, we measured the transcription of promoter-*lacZ* fusions containing various lengths of the P*gadE* region in Δ*arcA* and Δ*mnmE* strains, respectively (Figure 4). Surprisingly, deletion of *arcA* (grid bars) or *mnmE* (gray bars) caused decrease of transcription from the full length and all the truncation mutants with variable promoter lengths, indicating ArcA and MnmE might affect transcription from multiple promoters, especially the P3 and P1 regions, since P2/P4 and P individual promoters were not active under this condition as shown above. Considering that the activation of the P1 promoter is dependent on GadE, it is possible that ArcA and MnmE primarily activate the P3 promoter.

**Figure 5:**
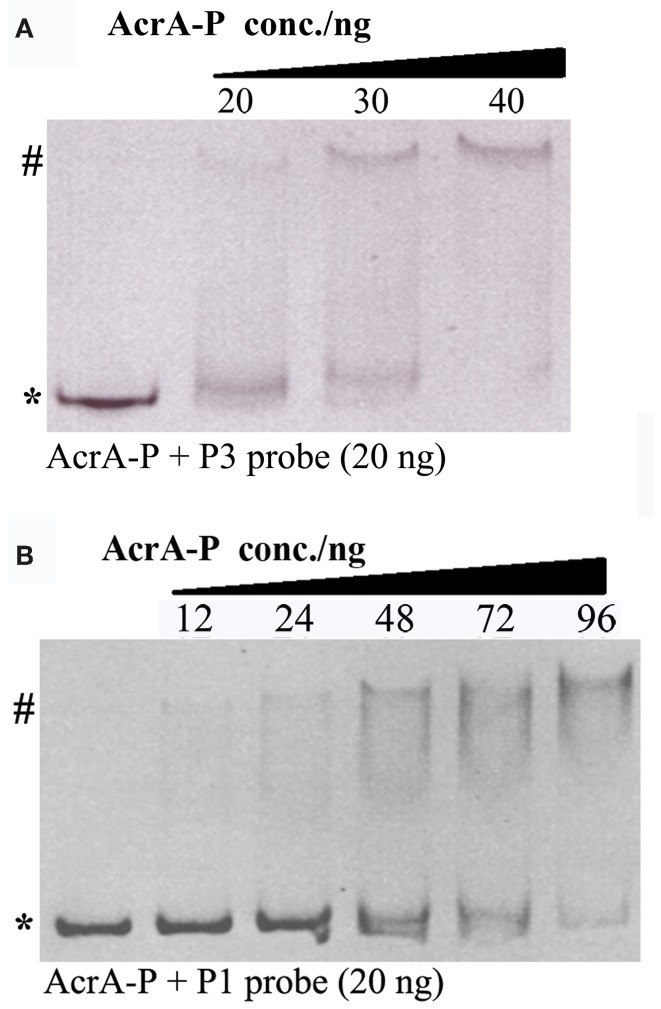
**Binding of ArcA-P to P1 and P3 promoters using EB stained EMSA assay**. Various concentrations of AcrA-P were incubated with P3 **(A)** or P1 **(B)**. AcrA-P refers to phosphorylated His_6_-ArcA protein. Asterisk indicates free DNA probe and pound indicates protein–DNA complex.

To examine whether the ArcA dependent activation of P*gadE* is through its direct binding on the *gadE* promoter region, we performed EMSA to measure the direct binding of ArcA-P (phosphorylated His_6_-ArcA) to the P1 and P3 promoters. As shown in Figure 5, retarded bands corresponding to the DNA-protein complexes of ArcA-P with both P3 and P1 were observed, suggesting AcrA-P binds to multiple sites at the *gadE* promoter encompassed in the individual P1 and P3 promoters and activates the expression of *gadE* under anaerobic condition. Genome wide CHIP-chip (Chromatin immunoprecipitation with microarray) and CHIP-seq study which determines all the ArcA binding sites across the *E. coli* K-12 MG1655 genome under anaerobic conditions in glucose minimum media also identified two broad ArcA binding peaks in the *gadE* promoter. Furthermore, the presence of the ArcA peaks correlated with the presence of a strong σ^70^ peak comparing with the very low σ^70^ peak on this region under aerobic condition (Park and Kiley, et al., unpublished data, pers. communication), further confirming the ArcA dependent activation of the *gadE-mdtEF* operon through its direct binding of multiple sites on the *gadE* promoter.

### ArcA antagonizes the H-NS mediated repression of *gadE*-*mdtEF* during the transition from aerobic to anaerobic growth

**Figure 6:**
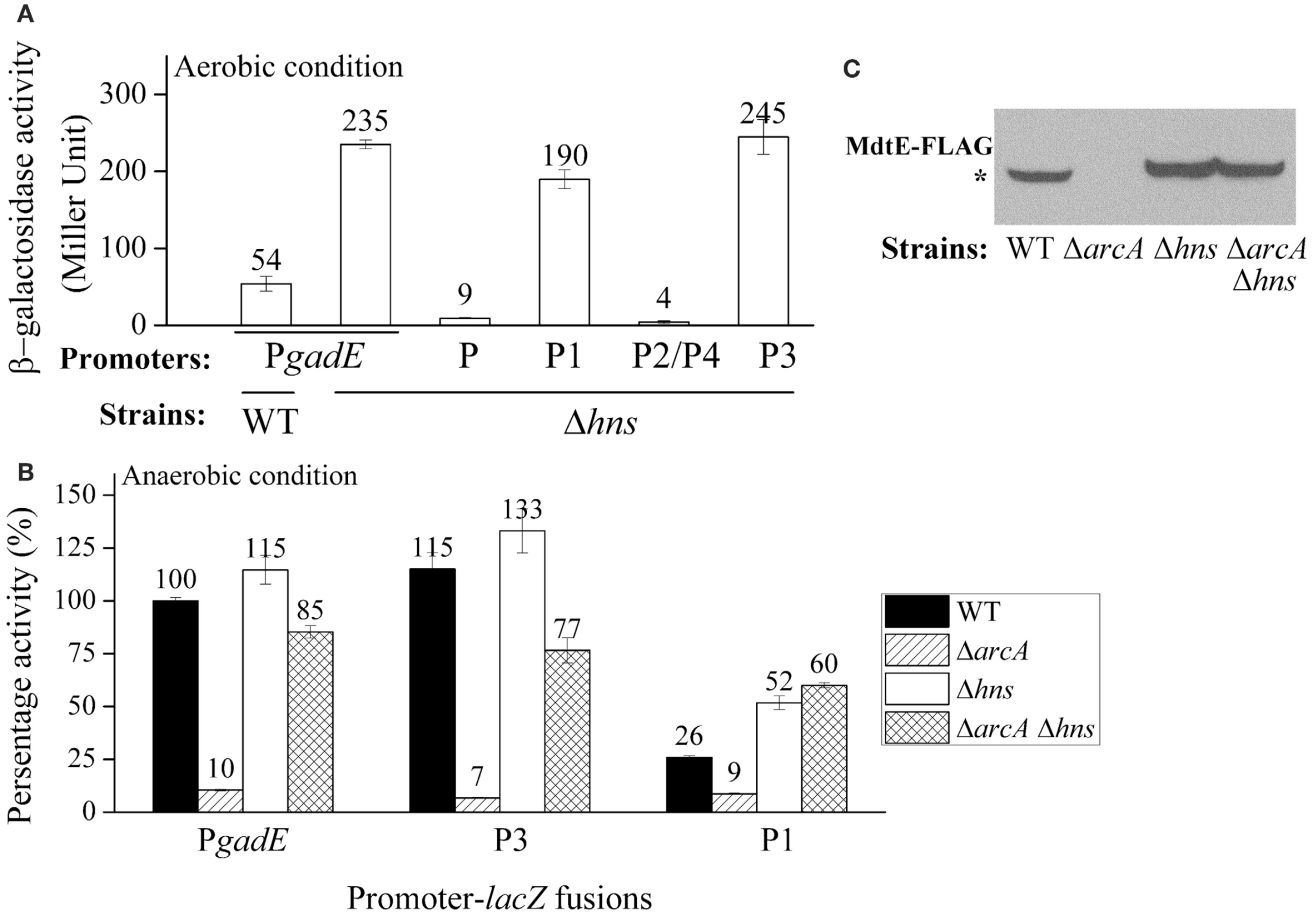
**ArcA antagonizes the H-NS mediated repression of *gadE* under anaerobic growth. (A)** Promoter activity of P*gadE* and its truncations under aerobic condition determined by β-galactosidase activity of corresponding *lacZ* fusions in WT and Δ*hns* strains; **(B)** Promoter activity of P*gadE* and its truncations under anaerobic condition determined by β-galactosidase activity of corresponding *lacZ* fusions in WT, Δ*arcA*, Δ*hns*, and Δ*arcA* Δ*hns* strains; **(C)** Protein level of MdtE-FLAG in WT, Δ*arcA*, Δ*hns* and Δ*arcA* Δ*hns* strains determined by Western blot. Cells used for all assays were grown anaerobically in M9 minimal glucose medium to exponential phase (A_600_ = 0.3). Results of β-galactosidase activity assay are presented in Miller unit or percentage activity to that of wild type strain, error bars represent the standard errors of triplicate experiments (*n* = 3). ^*^ indicates the band of MdtE-FLAG.

Our results above showed that the expression of *gadE-mdtEF* is largely repressed by the DNA silencer H-NS under aerobic conditions in M9 glucose medium, whereas under anaerobic condition in the same medium its expression is activated primarily in an ArcA dependent manner. This led us to ask how the H-NS mediated repression is replaced by the ArcA dependent activation during the transition of the facultative bacterium *E. coli* from its aerobic to anaerobic growth. To answer this question, we first examined which individual promoter region is responsible for the H-NS mediated repression under aerobic condition. As shown in Figure 6A, in addition to the full length promoter P*gadE*, deletion of *hns* caused de-repression of transcription from the P1 and P3 promoters, but no effect on the transcription from the P2/P4 and P promoters, indicating that H-NS represses the transcription of P*gadE* from both the P1 and P3 promoters under aerobic condition in the M9 glucose medium. This pattern is similar to that of how ArcA achieves its activation of P*gadE* under anaerobic condition which is also through its interaction with the P3 and P1 promoters.

Two possibilities may explain the transition from H-NS mediated repression to ArcA mediated activation of *gadE*-*mdtEF* during the transition from aerobic to anaerobic growth. One possibility is that H-NS is inactive under anaerobic conditions and it disassembles from the *gadE* promoter, allowing ArcA which is active under this condition to bind the promoter of *gadE* and initiate gene transcription. Alternatively, under anaerobic conditions H-NS can still bind to the *gadE* promoter, however, its binding is antagonized by the activated ArcA, resulting in the recruitment of RNAP and transcript initiation of the operon under this condition. To differentiate these two possibilities, we constructed Δ*arcA* Δ*hns* double deletion and examined transcription of P*gadE-lacZ* in this strain. If H-NS is inactive under anaerobic condition and is disassembled from the *gadE* promoter, then transcription of P*gadE*-*lacZ* in Δ*arcA* Δ*hns* should be similar to that of Δ*arcA* alone. However, if H-NS was not inactivated, rather, it can still bind to the promoter but its binding was antagonized by ArcA, then its binding to the *gadE* promoter should be resumed when ArcA is absent and an increase of P*gadE*-*lacZ* activity should be observed in Δ*hns* Δ*arcA* than Δ*arcA* single deletion strain. As shown in Figure 6B, transcription of P*gadE-lacZ* in Δ*arcA* Δ*hns* double deletion did display significantly higher activity than that in Δ*arcA* strain, suggesting that the anaerobically up-regulated *gadE-mdtEF* expression was through the antagonization of H-NS mediated repression by ArcA. Western blot analysis of chromosomal MdtE-FLAG showed the same pattern as that of transcription assay (Figure 6C). More interestingly, the increased transcription of *lacZ* fusions in Δ*arcA* Δ*hns* double deletion strain than that in Δ*arcA* single deletion strain was also observed in the case of individual P3 and P1 promoters, suggesting that ArcA antagonization of H-NS takes place at multiple sites in the P*gadE* promoter, consistent with the notion that both H-NS and ArcA can oligomerize and bind to a broad region in its regulated gene promoters (Green and Paget, 2004; Dorman, 2006). CHIP-chip data of H-NS and ArcA are also consistent with this proposal, as characterized by a broad peak of H-NS binding to PgadE under aerobic conditions and the replacement of this broad peak by that of ArcA under anaerobic conditions (Park and Kiley et al., unpublished data, pers. communication).

### The effect of MnmE on the anaerobic activation of *gadE-mdtEF* is not through ArcA

**Figure 7:**
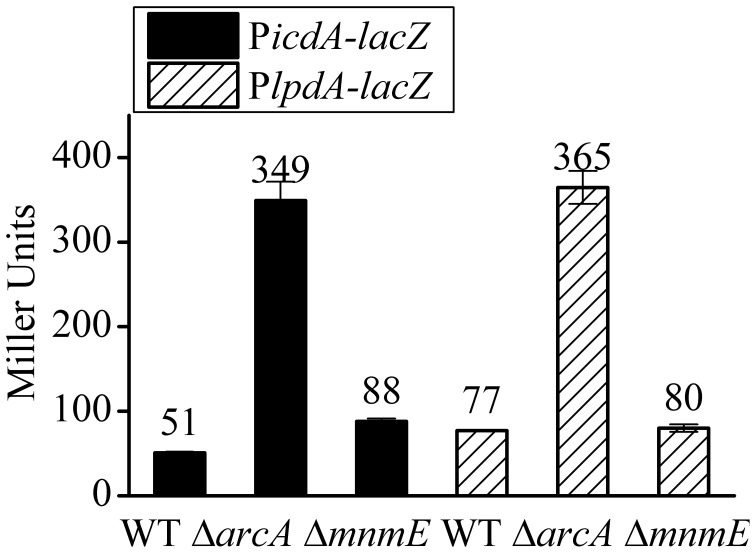
**MnmE has no effect on the transcription of P*icdA-lacZ* and P*lpdA-lacZ*, two ArcA dependent promoters**. β-galactosidase activities of chromosomal P*icdA*-*lacZ* (black bars) and P*lpdA-lacZ* (stripe bars) fusions were measured from cells grown anaerobically to exponential phase (A_600_ = 0.3) in minimal M9 glucose medium. Results are presented in Miller unit and error bars represent the standard errors of triplicate experiments (*n* = 3).

As shown in Figure 4, Δ*mnmE* caused a very similar pattern on the transcription activities of the various P*gadE* truncations as that caused by Δ*arcA*, only to a less extent. This, combined with the fact that MnmE itself does not have DNA binding property whereas ArcA was shown to directly bind to P*gadE* and activate its expression, led us to speculate that the effect of Δ*mnmE* on *gadE-mdtEF* expression may be indirect, i.e., MnmE may affect the translation and/or activity of ArcA (such as through affecting its phosphorylation). To address this, we tested the effect of Δ*mnmE* on the transcription of two ArcA dependent promoters, P*lpdA* and the mutated P*icdA*. Transcription of *lpdA* gene has been shown to be mediated by the repression of ArcA under anaerobic conditions. In the case of *icdA*, it contains two known promoters, P_I_ and P_II_, in which P_I_ is the primary promoter and is repressed only by ArcA. In the mutated P*icdA-lacZ*, transcription from P_II_ was eliminated by removal of its −10 element such that transcription repression of this P*icdA-lacZ* is exclusively ArcA dependent (Park and Kiley et al., unpublished work, pers. communication). If MnmE affects either the production or phosphorylation of ArcA (which affects the regulatory activity of ArcA), Δ*mnmE* should cause full or partial de-repression of these two promoters under anaerobic condition. As shown in Figure 7, as expected, transcription of P*icdA*-*lacZ* and P*lpdA-lacZ* fusions were significantly repressed by ArcA under anaerobic condition, since Δ*arcA* caused de-repression of their transcription under anaerobic condition in M9 glucose medium, the same condition used in this study. However, Δ*mnmE* had little or no effect on the transcription of these two promoters, suggesting that MnmE does not affect the production or phosphorylation of ArcA *in vivo*. Therefore, the observed MnmE mediated anaerobic activation of *gadE-mdtEF* was not due to its potential effects on ArcA.

## Discussion

### Different regulatory circuits on the expression of the *gadE-mdtEF* operon under different physiological conditions

Owing to the important roles of GadE and MdtEF in acid resistance and drug efflux respectively in *E. coli*, transcriptional regulation of the *gadE-mdtEF* operon under various of growth phases and conditions has been extensively investigated. While previous studies largely focused on its relevance in bacterial acid resistance under aerobic growth environment, the current study aimed to dissect the up-regulation of the operon under the physiologically relevant environment of anaerobic growth and its effect on the production of the multidrug efflux protein MdtEF.

Several well-characterized regulatory circuits that activate *gadE* expression in the context of acid resistance have been disclosed. These include the EvgA-YdeO-GadE feed forward regulatory loop which is effective in minimal glucose medium under aerobic condition (Ma et al., 2004), the conditional activation of the operon in a MnmE dependent manner in LB containing glucose (LBG) (Gong et al., 2004), as well as the GadX or GadW dependent activation which functions primarily in rich media in stationary growth phase (Sayed et al., 2007). Here we show that the anaerobic activation of the operon, particularly the expression of the multidrug efflux protein MdtEF, is also dependent on the 798 bp *gadE* promoter and the activation is primarily dependent on the anaerobic global regulator ArcA, adding yet another regulatory circuit to the existing complex regulatory loops of the operon. These results reinforced the important role of the promoter region of *gadE* in integrating various of environmental and physiological signals to facilitate the adaptation of *E. coli*.

We also measured the transcription of *PgadE-lacZ* under anaerobic condition in stationary phase and examined whether the previously identified regulators, such as RpoS and GadE, participate in the regulation of the *gadE-mdtEF* expression under this growth phase anaerobically. Surprisingly, while a slightly higher transcription of *PgadE-lacZ* in stationary phase than in log phase (~700 Miller units vs. ~480 Miller units in log phase) was observed, deletion of *rpoS* or *gadE* did not have any effect on the transcription of *PgadE-lacZ* in this growth phase (data not shown), whereas *ΔarcA* still caused significant decrease of the *gadE-mdtEF* expression in the same growth phase. These results suggested that anaerobic expression of the *gadE-mdtEF* operon is primarily regulated by ArcA in both log phase and stationary phase under anaerobic condition. It is noted that previously a putative ArcA binding site located at the P2 region was proposed (Zhang et al., 2011), whereas in the current study, the P3 and P1 region of the *gadE* promoter was shown to be bound by ArcA and they play an important role in the anaerobic activation of the operon. This inconsistency is likely due to the inaccuracy of the bioinformatic search which utilized a sequence motif of “(5′ [A/T]GTTAATTA[A/T] 3′)” as the template and the motif was proposed as the ArcA binding site by Lynch and Lin (1996) following the DNA foot-printing analysis of a limited number of ArcA regulated promoters. Indeed, a genome-wide search of all the ArcA regulated promoters and subsequent characterization of them suggest a motif (Kiley et al., unpublished work, pers. communication) that deviates from the previously proposed ArcA binding site.

**Figure 8:**
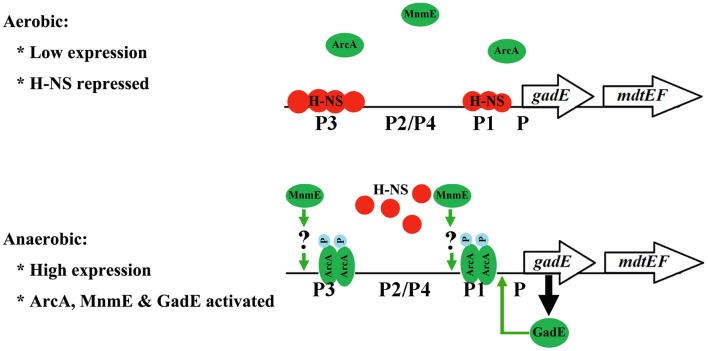
**A regulatory model for the transcriptional regulation of *gadE-mdtEF* operon during the transition from aerobic to anaerobic growth in minimal M9 glucose medium**. During aerobic growth, the *gadE-mdtEF* is expressed at a very low level due to the strong repression by H-NS. Upon transition to anaerobiosis, the response regulator ArcA is phosphorylated and activated by its sensor kinase ArcB. Phosphorylated ArcA (ArcA-P) antagonizes H-NS binding to *gadE* promoter, resulting in the relief of repression. ArcA-P also directly binds to the *gadE* promoter and activates the expression of *gadE-mdtEF*. ArcA-P activated *gadE* expression produces GadE, which activates the transcription from the P1 promoter and further up-regulate the *gadE-mdtEF* expression. MnmE also participates in the anaerobic activation of *gadE-mdtEF*, but in a yet uncharacterized manner.

Interestingly, we demonstrated that the ArcA dependent activation of the operon is through its antagonistic effect on the H-NS mediated repression of the operon. This regulatory model explains the dynamic process taking place during the transition of *E. coli* from its aerobic to anaerobic growth, which mimics the transition of the bacterium from free living status to the anaerobic environment of the human host. H-NS mediated repression of the operon has also been implicated in previous studies (Ma et al., 2004; Sayed and Foster, 2009). However, the inter-relationship of this repression and gene activation mediated by other regulators has not been revealed. Our findings demonstrating that ArcA activates the expression of the operon through antagonizing the H-NS mediated repression probably provide a common model that underlies the dynamic and coordinated regulation of *gadE-mdtEF* in response to different environmental signals. Perhaps under unstressed conditions when the expression of the operon is unnecessary, the promoter of *gadE* is primarily occupied by H-NS and transcription from the various promoters in this region is repressed. Upon the activation of different transcription activators in response to different environmental or physiological conditions that presage acid stresses or efflux of certain metabolic by-products, the activators then displace H-NS binding on the promoter, resulting in the activation of the operon. Several lines of evidence support this proposal: first, bioinformatics have suggested the presence of an efficient DNA helix locking mechanism by H-NS in the promoter of *gadE* (Hommais et al., 2004); Second, H-NS mediated repression has been found to be common among acquired gene clusters which is consistent with the functions of these gene clusters in conferring the survival and adaptation of bacteria in various adverse growth environments (Oshima et al., 2006). The acid resistant island including the *gadE-mdtEF* operon indeed is one of the acquired gene clusters in *E. coli* with different GC content from the rest of *E. coli* genome; Third, the interplay between the H-NS mediated repression and ArcA mediated antagonizing of the repression is also observed in the case of another gene *cydAB* which is also involved in adapting *E. coli* to the microaerobic and anaerobic growth (Govantes et al., 2000). Thus, anaerobic activation of *gadE-mdtEF* through antagonizing the H-NS mediated repression by ArcA provides a regulatory model that is relevant to the dynamic lifestyle of the facultative bacterium *E. coli*. A model to explain this dynamic regulation is presented in Figure 8.

### Roles of the *gadE-mdtEF* operon in both acid resistance and drug efflux

It is interesting that the drug efflux genes *mdtEF* exist in the same operon with the central acid resistance regulator *gadE*, and their expression is driven by the same unusually long 798 bp promoter region upstream of the *gadE* gene. While functions of GadE in controlling the expression of the major acid resistant system GadA and GadBC is well established, the relevance of MdtEF to acid resistance has not been fully recognized. Our previous studies showed that MdtEF functions to expel indole nitrosative by-products accumulated during nitrate respiration under anaerobic condition (Zhang et al., 2011). It is known that anaerobic metabolism often causes pH reduction in growth medium, especially during glucose fermentation by *E. coli* which leads to the production of various organic acids. Although anaerobic induction of *gadE-mdtEF* occurs at a fairly neutral pH (6.9 at the log phase culture when the cells were harvested for transcription and Western blot analyses), significant accumulation of the indole nitrosative by-product occurred when pH dropped to 5.8 or below (Weiss, 2006). Thus, it seems that MdtEF does contribute to the detoxification of acid induced metabolic by-products. Yet, its activation does not necessarily depend on the occurrence of acid stresses. This is consistent with the findings from previous studies which propose that the *gadE-mdtEF* operon is rigged to be induced under many different circumstances that could presage an encounter with acid stresses (Hommais et al., 2004).

This raises the interesting question of whether the housekeeping pump AcrAB is also involved in the nitrosative and/or acid resistance by expelling metabolic by-products under anaerobic conditions. Although the broad substrate specificity of AcrAB-TolC was established in terms of its capability of exporting antibiotics, we speculate that the same degree of broad substrate specificity may not apply in terms of its capability of exporting physiological substances, since it has been well recognized that expression of certain efflux pump genes other than the housekeeping AcrAB pump is activated by specific environmental or physiological conditions (Piddock, 2006; Li and Nikaido, 2009; Poole, 2012b). We recently performed metabolic profiling of the WT vs. *ΔmdtEF* under the growth condition of anaerobic nitrate respiration and it was shown that *ΔmdtEF* cells accumulated more indole nitrosative by-products than the WT (data not shown) in the presence of the *acrAB* genes, suggesting that the MdtEF pump exhibits at least certain degree of substrate specificity even in the presence of the housekeeping pump AcrAB. It is highly likely that MdtEF also expels other metabolic by-products accumulated during acid stresses. If that is the case, there are probably more extensive relevance between MdtEF mediated drug efflux and the physiology of *E. coli* during acid resistance. This relevance probably can be disclosed through metabolic profiling by comparing the total metabolites in Δ*mdtEF* and WT strain grown during acid stresses.

### The unresolved role of MnmE in the up-regulation of *mdtEF* expression

MnmE is a GTP binding protein that is involved in tRNA modification (Cabedo et al., 1999). This protein was found to be in cytoplasm but can partially associate with the inner membrane (Cabedo et al., 1999). Its involvement in the conditional activation of *gadE* at exponential growth in LB medium with glucose (LBG) or stationary growth of *E. coli* in unbuffered LBG under aerobic condition has been reported (Gong et al., 2004). However, the detailed regulatory mechanism remains elusive. Since MnmE itself does not have DNA binding property, it was speculated that MnmE affects the *gadE* expression by controlling an unknown regulator which can bind to *gadE* promoter and mediate its transcription. Since Δ*mnmE* resulted in a similar pattern of decreased transcription from both full length and various truncation promoter fusions as in Δ*arcA* strain except to a less extent, and ArcA was shown to directly bind to P*gadE*, we had speculated that MnmE regulation of the *gadE* expression was through its effect on ArcA, i.e., through its mediation of the translation and/or phosphorylation of ArcA. However, our β-galactosidase activity results showing that Δ*mnmE* does not have any effect on two different ArcA dependent promoters (P*icdA* and P*lpdA*) suggested that MnmE does not affect the production and/or activity of ArcA under anaerobic conditions, and thus the observed reduction of *gadE-mdtEF* expression in Δ*mnmE* strain was not due to its potential effect on ArcA.

Studies also indicated that MnmE dependent conditional expression of *gadE* mainly depends on the P2 promoter region (Sayed and Foster, 2009). However, our study showed that the P2 region is not active under anaerobic condition at log growth phase; instead, the MnmE dependent *gadE* activation was likely through its effect on the P3 region, which is indispensable in the anaerobic activation of *gadE*. Furthermore, we found that MnmE dependent activation of the *gadE-mdtEF* expression also occurred in non-glucose media, such as LB and glycerol supplemented with nitrate (data not shown). This observation is not in agreement with a previous finding which suggested that MnmE dependent *gadE* expression was induced only in the presence of glucose. Thus, although several studies support the notion that the GTPase MnmE participates in the transcriptional activation of the *gadE-mdtEF* operon under certain growth conditions and phases, how exactly MnmE is involved in the process is still ambiguous. This is largely due to the fact that the exact biological function of MnmE itself is still elusive. Obviously, further studies of MnmE protein is needed before we can reveal its role in the up-regulation of *gadE-mdtEF* under the physiologically relevant conditions.

It is noteworthy that the 798 bp sensory integration region of the *gadE-mdtEF* promoter is highly conserved among the pathogenic *E. coli* strains, including pathogenic strains O157:H7, 2369, RS128 and CFT073 and Shigella (96–99% identity), and GadE has been demonstrated to be indispensable for the survival of *E. coli* O157::H7 in a simulated gastric environment (Kailasan Vanaja et al., 2009), underlying the importance of the *gadE-mdtEF* operon to the physiology of *E. coli*. Since both low pH, such as that of gastric acid, and oxygen limitation (anaerobic environment) activates the expression of the *gadE-mdtEF* operon, it is conceivable that synergetic activation of the system by both regulatory circuits may occur during the transition of *E. coli* to human host, particularly the passage of the gastrointestinal tract, thus greatly facilitate the adaptation and survival of this neutrophilic bacterium in its host niches.

### Conflict of interest statement

The authors declare that the research was conducted in the absence of any commercial or financial relationships that could be construed as a potential conflict of interest.
